# Identification and validation of *HOXB3* hypomethylation as a novel prognostically epigenetic biomarker in acute myeloid leukemia

**DOI:** 10.3389/fimmu.2025.1709417

**Published:** 2026-01-12

**Authors:** Ting-juan Zhang, Ran Chang, Fei Xie, Zi-jun Xu, Ming-qiang Chu, Xiao-chi Wu, Jun Qian, Jing-dong Zhou

**Affiliations:** 1 Department of Hematology, The Affiliated People’s Hospital of Jiangsu University, Zhenjiang Clinical Research Center of Hematology, Zhenjiang, Jiangsu, China; 2 Institute of Hematology, Jiangsu University, Zhenjiang, Jiangsu, China; 3 The Key Lab of Precision Diagnosis and Treatment in Hematologic Malignancies of Zhenjiang City, Zhenjiang, Jiangsu, China; 4 Department of Radiation Oncology, The Affiliated People’s Hospital of Jiangsu University, Zhenjiang, Jiangsu, China

**Keywords:** AML, HOXB, HoxB3, hypomethylation, prognosis, regulation

## Abstract

**Background:**

Aberrant expression of Homeobox (*HOX*) genes has been observed in acute myeloid leukemia (AML), but their epigenetic regulatory mechanisms remain largely elusive. Previously, we identified *HOXA9* hypomethylation, among *HOXA* family genes as an epigenetic biomarker for predicting clinical outcomes and guiding treatment choices in AML. Herein, we further investigated the methylation of *HOXB* family members in AML and determined its clinical implications.

**Methods:**

We first systematically analyzed the association of *HOXB* methylation with expression and clinical outcomes in AML from The Cancer Genome Atlas (TCGA) database. Next, the candidate prognosis-related gene *HOXB3* was selected for clinical relevance analysis and further verified in another independent cohort from our hospital.

**Results:**

Hypomethylation of *HOXB3*, which was negatively associated with its expression, was correlated with adverse prognosis among *HOXB* family genes in AML from TCGA datasets. Clinically, AML patients with *HOXB3* hypomethylation had unique clinical subtypes and cytogenetic/molecular patterns, including FAB-M5, normal karyotype, cytogenetic/molecular-intermediate risks, and mutations in *FLT3*-ITD, *NPM1* and *DNMT3A*. Despite these associations, *HOXB3* hypomethylation was an independent prognostic biomarker for AML. Bioinformatics analysis demonstrated the association of *HOXB3* hypomethylation with several leukemia-related genes (*HOXB* family genes, *miR-10*, *miR-196a*, *miR-1*, *miR-193b* and *miR-379*) in AML. Subsequently, we further validated aberrant *HOXB3* hypomethylation and its epigenetic regulatory role in AML.

**Conclusions:**

*HOXB3* hypomethylation, which is associated with *HOXB3* overexpression, is a frequent event in AML. AML with *HOXB3* hypomethylation usually has unique genetic patterns such as a normal karyotype, cytogenetic/molecular-intermediate risk, and mutations in *FLT3*-ITD, *NPM1* and *DNMT3A*. Despite these associations, *HOXB3* hypomethylation may serve as an independent prognostic biomarker for AML.

## Introduction

Acute myeloid leukemia (AML) is a heterogeneous aggressive blood cancer with an unsatisfactory prognosis and 5-year overall survival (OS) rate of less than 50% (1). Genetic alterations and epigenetic modifications lead to abrogated differentiation, blocked apoptosis, and uncontrolled proliferation of hematopoietic stem cells (1). Over the past two decades, we have gained deeper insights into the roles of cytogenetics and molecular biology in the pathogenesis and progression of AML (1, 2). In recent years, epigenetic modifications, such as aberrant DNA methylation, a major biological process in silencing the transcription of tumor suppressor genes, have contributed to the pathophysiology of myeloid malignancies and hypomethylating agents are commonly used to treat AML and myelodysplastic syndromes/neoplasm (MDS) (3).

Homeobox (*HOX*) genes belong to a family of homeodomain containing transcription factors that are mainly involved in early development and hematopoiesis (4). A total of 39 members have been identified in the human *HOX* gene family, which is divided into four families: *HOXA*, *HOXB*, *HOXC*, and *HOXD* (4). *HOXA* family genes, particularly *HOXA9* have been thoroughly studied in AML (5). Aberrant methylation was preferentially found in *HOXA* gene sequences, including *HOXA1*, *HOXA4*, *HOXA5*, *HOXA9*, *HOXA10*, and *HOXA11* which affect their expression (6). Moreover, our previous study also identified *HOXA9* hypomethylation in *HOXA* family as an epigenetic biomarker for predicting prognosis and guiding treatment choice in AML (7). Among *HOXB* family, although some studies have suggested that several *HOXB* genes are upregulated in certain types of AML (8–10), their epigenetic dysregulation in AML has not yet been elucidated. Following our previous study, we systematically investigated methylation of *HOXB* family members in AML and determined their clinical significance.

## Materials and methods

### Public datasets

This study included a cohort of 200 AML patients from The Cancer Genome Atlas (TCGA) public databases (11). Clinical data and laboratory findings of AML patients were downloaded from the cBioPortal (https://www.cbioportal.org/) (12). *HOXB* family gene expression data was available for 173 patients, whereas *HOXB* family gene methylation data was available for 194 patients (170 overlapping patients with both *HOXB* expression and methylation).

### Patients

A total of 54 *de novo* AML patients were also included in this research as the validation cohort. Clinico-pathological features of the patients were presented in **Supplementary Table S1**. After informed consents were obtained from all participants, bone marrow (BM) samples were collected and were prepared for BM mononuclear cells (BMMNCs) as reported (13, 14). Twenty healthy individuals served as the controls.

### Cell lines and cell culture

Human leukemic cell lines (KASUMI-1, OCI-AML-2, OCI-AML-3, THP-1, MV4-11, SKM-1, NOMO-1, HEL and DAMI) and BM stoma cell line (HS-5) were used in this study. Cell culture was performed as previous described (7).

### Hypomethylation agent and demethylation studies

The KASUMI-1 and OCI-AML-2 cells in 4 mL (5×10^5^ cells/mL) were treated with the hypomethylation agent 5-aza-2’-deoxycytidine (5-aza-dC) at a final concentration of 0 μM (control), 2 μM, 4 μM, and 10 μM for 3 days. All the cell lines were cultured until they are harvested for RNA and DNA extraction.

### RNA isolation, reverse transcription and RQ-PCR

Total RNA was isolated from cells using TRIzol reagent, followed by reverse transcription for cDNA synthesis as previously reported (13, 14). Real-time quantitative PCR (RQ-PCR) was conducted to examine the expression levels of *HOXB3* by the usage of TB Green^®^ Premix Ex Taq™ II (Tli RNaseH Plus) (RR820A, TaKaRa, Tokyo, Japan). The primers used for the detection of *HOXB3* expression were 5’-CTTGGACCGGCTGTTGG-3’ (Forward) and 5’-TTGTCGTAGTAGGTGGCTTT-3’ (Reverse). The RQ-PCR conditions were as follows: 95°C for 5 min, 40 cycles for 10 s at 95°C, 30 s at 56°C, 1 min at 72°C. The reference gene *GAPDH* was used as our previous literature (7). The relative *HOXB3* expression levels were measured using 2^-ΔΔCt^ method.

### DNA isolation, bisulfite modification, and RQ-MSP

Genomic DNA was extracted from the cells using a Puregene Blood Kit (158023, QIAGEN, Duesseldorf, Germany), followed by bisulfite modification with the usage of EZ DNA Methylation-Gold^™^ Kit (D5002, ZYMO RESEARCH, Orange County, California). Real-time quantitative methylation-specific PCR (RQ-MSP) was performed to detect the methylation level of *HOXB3* by the usage of TB Green^®^ Premix Ex Taq™ II (Tli RNaseH Plus) (RR820A, TaKaRa, Tokyo, Japan). The primers utilized for the detection of *HOXB3* methylation were 5’-TTTCGGATCGTTTATACGC-3’ (Methylation Forward) and 5’-CACTTCATACGCCGATTCTA-3’ (Methylation Reverse) as well as 5’-GTTTTTGGATTGTTTATATGT-3’ (Unmethylation Forward) and 5’-CCACTTCATACACCAATTCTAA-3’ (Unmethylation Reverse). The conditions of RQ-MSP program were 95°C for 5 min, 40 cycles for 10 s at 95°C, 30 s at 56°C (Methylation primers) or 54 °C (Unmethylation primers), 30 s at 72°C, 30s at 75 °C. The reference gene *ALU* was detected as our previous literature (7). The Relative *HOXB3* methylation levels were calculated using the 2^-ΔΔCT^ method.

### Bioinformatics analysis

The detailed bioinformatics analysis referred to our previous article (7).

### Statistical analysis

SPSS 22.0 and GraphPad 8.0 software were used for the statistical analysis. The Mann-Whitney U-test/Kruskal-Wallis test/Student’s T-test and Pearson’s χ^2^-test/Fisher’s exact test were used to compare the continuous and categorical variables, respectively. Kaplan-Meier method (log-rank test) and Cox regression analysis (proportional hazards) were performed to determine the prognostic impact of *HOXB* family genes methylation on OS and disease-free survival (DFS). The receiver operating characteristic (ROC) curve and area under the ROC curve (AUC) were conducted to evaluate the ability of *HOXB3* methylation to distinguish patients with AML from controls. A two-sided *P* value less than 0.05 was considered statistically significant.

## Results

### Identification of HOXB3 hypomethylation among HOXB family genes associated with prognosis with expression in AML

**Figure 1:**
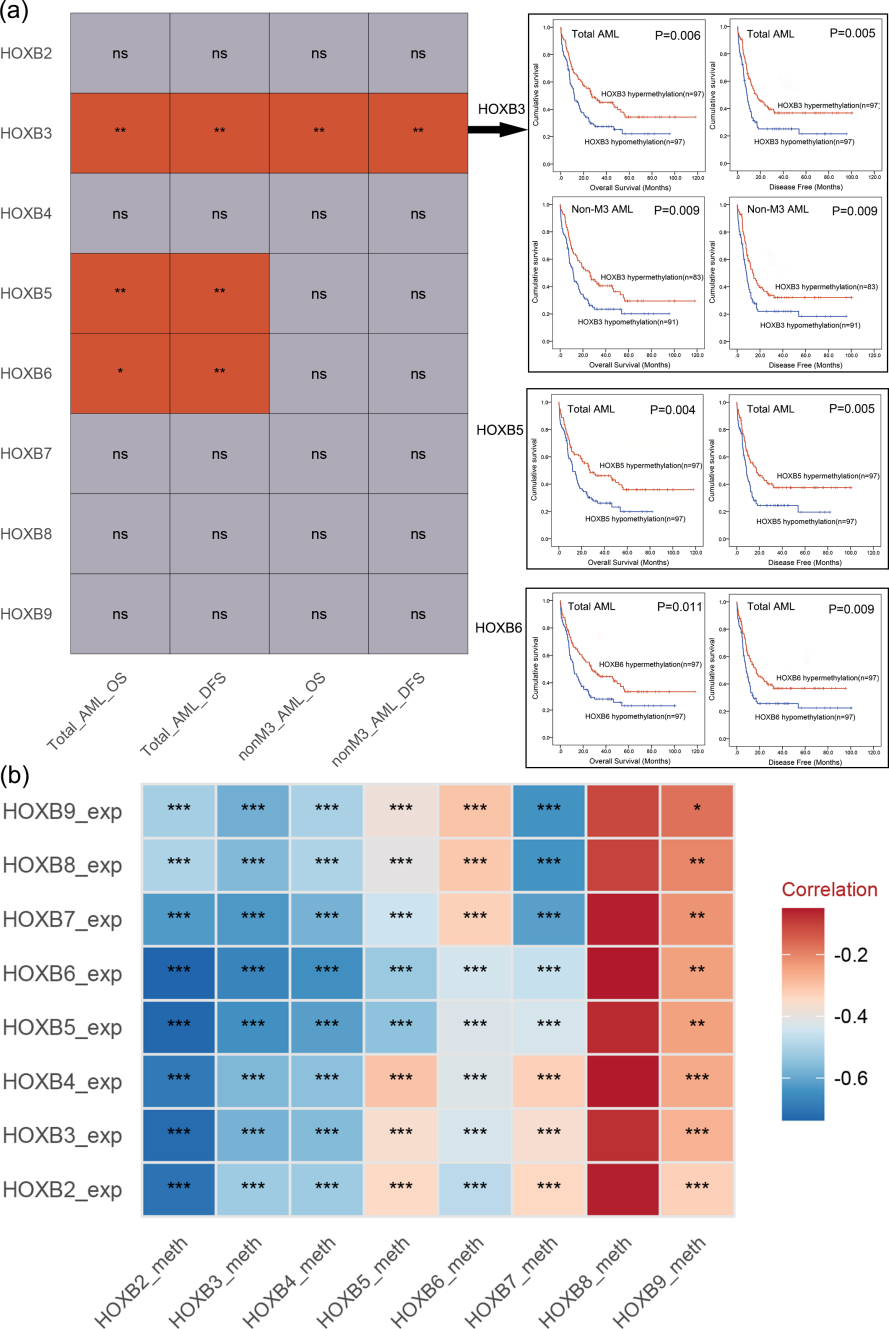
Identification of *HOXB* family genes methylation correlated with prognosis and expression in AML. **(a)** The statistical value of the prognostic significance of each *HOXB* family gene methylation on survival in AML patients (determined by Kaplan-Meier analysis). Moreover, the survival curve of the impact of *HOXB3*, *HOXB5* and *HOXB6* methylation on overall/disease-free survival in total/non-M3 AML patients with significant difference were also presented. Total_AML_OS: The prognostic effect of each *HOXB* family gene methylation on overall survival among total AML; Total_AML_DFS: The prognostic effect of each *HOXB* family gene methylation on disease free survival among total AML; nonM3_AML_OS: The prognostic effect of each *HOXB* family gene methylation on overall survival among non-M3 AML; nonM3_AML_DFS: The prognostic effect of each *HOXB* family gene methylation on disease free survival among non-M3 AML. NS, no significant; *P < 0.05; **P < 0.01. **(b)** The statistical value of the correlation analysis between each *HOXB* gene expression and methylation in AML patients (determined by Spearman test). The depth of color differences represents correlation coefficients. *P < 0.05; **P < 0.01; ***P < 0.001.

To identify the crucial epigenetic markers in *HOXB* family in AML, we first analyzed the prognostic value of *HOXB* members methylation with available data (*HOXB2*/*HOXB3*/*HOXB4*/*HOXB5*/*HOXB6*/*HOXB7*/*HOXB8*/*HOXB9*) using TCGA databases. The patients were divided into two groups (hyper- and hypo- methylation) based on the median methylation level of each gene. In total AML patients, hypo-methylation of *HOXB3*, *HOXB5* and *HOXB6* was markedly correlated with shorter OS (*P* = 0.006, 0.004 and 0.011, respectively) and DFS (*P* = 0.005, 0.005, and 0.009, respectively), whereas the other members did not affect survival (both *P*>0.05, **Figure 1a**). As patients with AML-M3 have extremely favorable outcomes, we further explored the prognostic impact of these members in non-M3 AML. Notably, only *HOXB3* hypo-methylation had a significant prognostic effect on OS and LFS (both *P* = 0.009, **Figure 1a**).

We further tested the prognostic effect of *HOXB* expression on OS and LFS in AML patients using Cox regression analysis. In all AML patients, none of the *HOXB* members had prognostic effects on OS or DFS (all *P*>0.05, **Table 1**). However, among non-M3 AML patients, *HOXB3* hypo-methylation significantly affected the OS and DFS (both *P* = 0.009, **Table 1**).

**Table 1:** Univariate and multivariate Cox regression analysis of *HOXB* genes methylation for overall survival and leukemia-free survival in AML patients.

Variables	Total AML				Non-M3 AML			
	Univariate analysis		Multivariate analysis		Univariate analysis		Multivariate analysis	
	HR (95% CI)	*P*	HR (95% CI)	*P*	HR (95% CI)	*P*	HR (95% CI)	*P*
Overall Survival								
*HOXB2* methylation	0.869 (0.615-1.229)	0.428			1.003 (0.703-1.432)	0.986		
*HOXB3* methylation	0.613 (0.432-0.869)	0.006	0.719 (0.487-1.064)	0.099	0.620 (0.433-0.890)	0.009	0.620 (0.433-0.890)	0.009
*HOXB4* methylation	0.929 (0.658-1.313)	0.676			0.999 (0.700-1.425)	0.995		
*HOXB5* methylation	0.603 (0.425-0.857)	0.005	0.700 (0.473-1.037)	0.075	0.746 (0.521-1.070)	0.112	0.965 (0.626-1.487)	0.872
*HOXB6* methylation	0.639 (0.451-0.906)	0.012	0.821 (0.542-1.244)	0.352	0.706 (0.493-1.012)	0.058	0.838 (0.563-1.247)	0.384
*HOXB7* methylation	0.813 (0.575-1.149)	0.241			0.844 (0.591-1.206)	0.352		
*HOXB8* methylation	0.992 (0.702-1.402)	0.966			1.138 (0.797-1.624)	0.477		
*HOXB9* methylation	0.943 (0.667-1.334)	0.741			0.817 (0.731-1.488)	0.817		
Disease-free Survival								
*HOXB2* methylation	0.855 (0.605-1.208)	0.375			0.983 (0.689-1.403)	0.925		
*HOXB3* methylation	0.608 (0.429-0.802)	0.005	0.698 (0.477-1.022)	0.064	0.621 (0.433-0.890)	0.009	0.621 (0.433-0.890)	0.009
*HOXB4* methylation	0.939 (0.665-1.326)	0.720			1.000 (0.701-1.427)	0.998		
*HOXB5* methylation	0.613 (0.432-0.869)	0.006	0.708 (0.483-1.036)	0.076	0.774 (0.541-1.109)	0.162	0.992 (0.649-1.516)	0.970
*HOXB6* methylation	0.633 (0.447-0.898)	0.010	0.803 (0.532-1.211)	0.295	0.713 (0.498-1.022)	0.065	0.837 (0.566-1.236)	0.371
*HOXB7* methylation	0.771 (0.546-1.090)	0.142	1.124 (0.736-1.717)	0.589	0.811 (0.568-1.158)	0.249		
*HOXB8* methylation	0.980 (0.694-1.385)	0.910			1.127 (0.790-1.609)	0.509		
*HOXB9* methylation	0.991 (0.702-1.400)	0.959			1.085 (0.761-1.548)	0.652		

HR, hazard ratio; CI, confidence interval; Multivariate analysis includes variables with *P* < 0.200 in univariate analysis.

Promoter-associated DNA methylation is a major mechanism regulating gene expression. We analyzed the association between *HOXB* methylation and expression in patients with AML. A significant negative correlation was observed for *HOX2*/*HOXB3*/*HOXB4*/*HOXB5*/*HOXB6*/*HOXB7* but not for *HOXB8*/*HOXB9* (**Figure 1b**).

Taken together, these results suggest that *HOXB3* hypo-methylation may be the most valuable prognostic biomarker among the *HOXB* genes in AML, especially in non-M3 AML, and was selected for further analysis.

### HOXB3 hypomethylation associated with specific subtypes of AML

To evaluate the clinical implications of *HOXB3* methylation in AML, we compared the clinico-pathological characteristics between *HOXB3* hypo- and hyper-methylation groups. There were no significant differences between the two groups in sex, age, white blood cell count, and peripheral blood (PB)/BM blast ratio (all *P*>0.05, **Table 2**). However, marked differences were observed between the two groups in the distribution of the French-American-British (FAB) classification (*P* = 0.009), karyotypic classification (*P* < 0.001), and molecular/cytogenetic risk classifications (all *P* < 0.001) (**Table 2**). Hypomethylation of *HOXB3* was significantly occurred in FAB-M5 (*P* = 0.011, **Table 2**). Among the karyotypic classification, *HOXB3* hypomethylation was more likely to occur in normal karyotype (*P* < 0.001), and rarely occurred in inv(16) (*P* < 0.001) (**Table 2**). As expected, hypomethylation of *HOXB3* was occurred markedly in intermediate karyotype and rarely occurred in good karyotype among both cytogenetic and molecular risk classifications (all *P ≤* 0.001, **Table 2**). Among common gene mutations, hypomethylation of *HOXB3* was associated with *FLT3* (*P* = 0.010), *NPM1* (*P* < 0.001), *DNMT3A* mutations (*P* < 0.001) (**Table 2**).

**Table 2:** Correlation of clinic-pathologic characteristics in AML patients with *HOXB3* hypo- and hyper-methylation.

Patient’s parameters	Total	Hypo- (n=97)	Hyper- (n=97)	*P* value
Sex, male/female	104/90	46/51	58/39	0.113
Median age, years (range)	57 (18-88)	60 (21-88)	55 (18-83)	0.100
Median WBC, ×10^9^/L (range)	16.6 (0.4-298.4)	19.9 (0.5-298.4)	16.0 (0.4-223.8)	0.071
Median PB blasts, % (range)	36 (0-98)	22 (0-98)	40.50 (0-97)	0.522
Median BM blasts, % (range)	73.5 (30-100)	75 (30-100)	71.5 (30-100)	0.283
FAB classifications				0.009
M0	19	5	14	0.051
M1	44	21	23	0.864
M2	42	24	18	0.384
M3	18	6	12	0.215
M4	41	22	19	0.725
M5	22	17	5	0.011
M6	3	2	1	>0.999
M7	3	0	3	0.246
No data	2	0	2	0.497
Karyotypes				<0.001
normal	91	63	28	<0.001
t(15;17)	16	6	10	0.435
t(8;21)	7	2	5	0.444
inv(16)	12	0	12	<0.001
+8	9	4	5	>0.999
del(5)	1	0	1	>0.999
-7/del(7)	8	4	4	>0.999
11q23	4	1	3	0.621
others	14	4	10	0.163
complex	28	11	17	0.307
No data	4	2	2	>0.999
Risks (cytogenetic)				<0.001
Good	35	8	27	0.001
Intermediate	112	70	42	<0.001
Poor	43	17	26	0.166
No data	4	2	2	>0.999
Risks (molecular)				<0.001
Good	37	8	29	<0.001
Intermediate	103	68	35	<0.001
Poor	51	20	31	0.102
No data	3	1	2	>0.999
Gene mutations				
*FLT3* (+/-)	55/139 (28.4%)	36/61 (37.1%)	19/78 (19.6%)	0.010
*NPM1* (+/-)	53/141 (27.3%)	47/50 (48.5%)	6/91 (6.2%)	<0.001
*DNMT3A* (+/-)	47/147 (24.2%)	40/57 (41.2%)	7/90 (7.2%)	<0.001
*IDH2* (+/-)	18/176 (9.3%)	8/89 (8.2%)	10/87 (10.3%)	0.805
*IDH1* (+/-)	19/175 (9.8%)	11/86 (11.3%)	8/89 (8.3%)	0.630
*TET2* (+/-)	16/178 (8.2%)	10/87 (10.3%)	6/91 (6.2%)	0.435
*RUNX1* (+/-)	15/179 (8.2%)	4/93 (4.1%)	11/86 (11.3%)	0.104
*TP53* (+/-)	16/178 (8.2%)	7/90 (7.2%)	9/88 (9.3%)	0.795
*NRAS* (+/-)	15/179 (8.2%)	7/90 (7.2%)	8/89 (8.2%)	>0.999
*CEBPA* (+/-)	13/181 (6.7%)	5/92 (5.2%)	8/89 (8.2%)	0.568
*WT1* (+/-)	11/183 (5.7%)	5/92 (5.2%)	6/91 (6.2%)	>0.999
*PTPN11* (+/-)	9/185 (4.6%)	6/91 (6.2%)	3/94 (3.1%)	0.497
*KIT* (+/-)	8/186 (4.1%)	3/94 (3.1%)	5/92 (5.2%)	0.721
*U2AF1* (+/-)	8/186 (4.1%)	3/94 (3.1%)	5/92 (5.2%)	0.721
*KRAS* (+/-)	6/188 (3.1%)	4/93 (4.1%)	2/95 (2.1%)	0.683

AML, acute myeloid leukemia; WBC, white blood cells; PB, peripheral blood; BM, bone marrow; FAB, French-American-British classification. Cytogenetic and molecular risk classifications are based on the 2017 European LeukemiaNet (ELN) classification.

**Figure 2:**
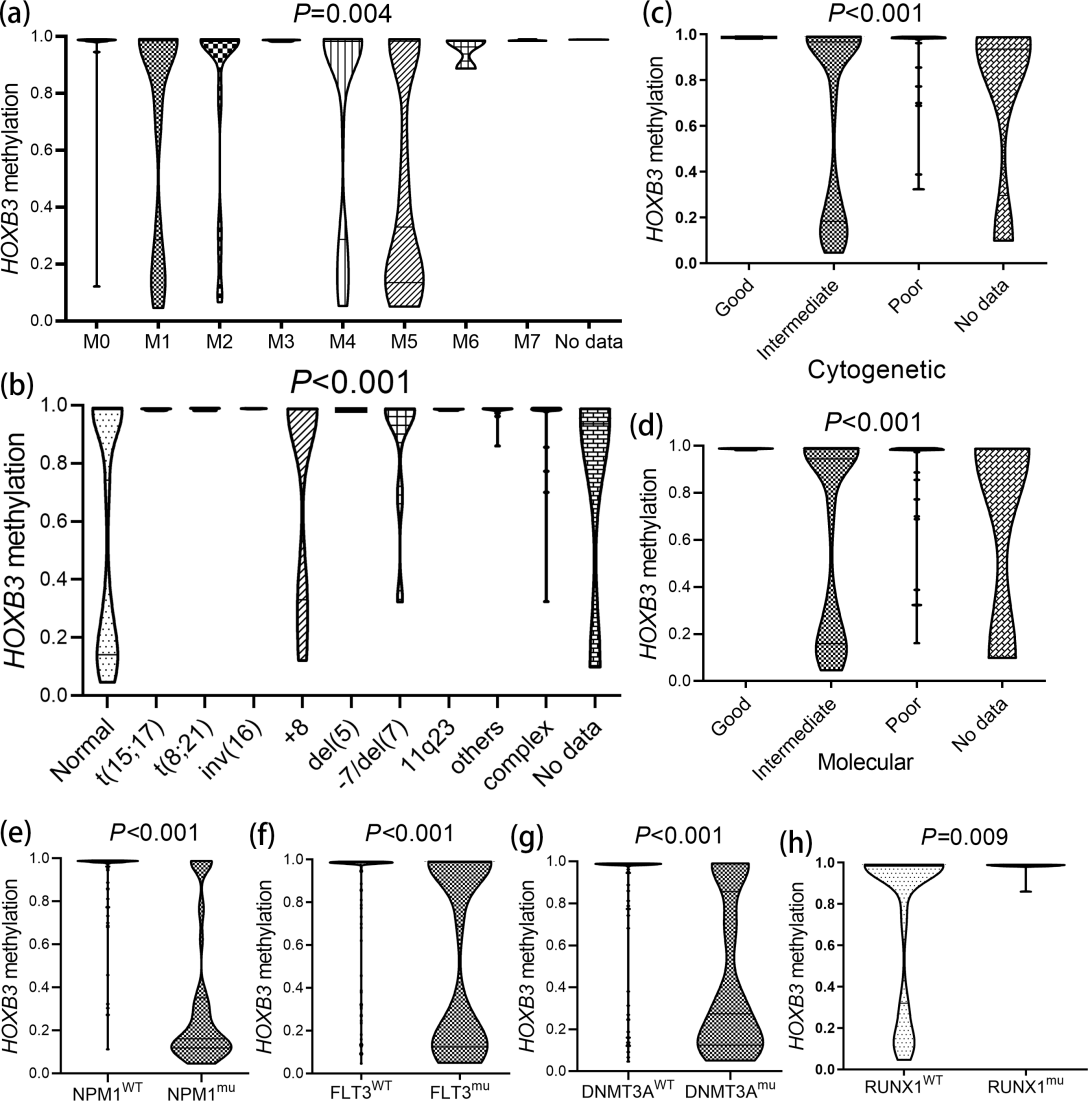
The associations of *HOXB3* methylation with specific subtypes of AML. **(a)***HOXB3* methylation level among different French-American-British (FAB) subtypes of AML. **(b)***HOXB3* methylation level among different cytogenetic risk subgroups of AML. **(c)***HOXB3* methylation level among different karyotypic subgroups of AML. **(d)***HOXB3* methylation level among different molecular risk subgroups of AML. **(e)***HOXB3* methylation level in groups of AML patients with and without *NPM1* mutations. **(f)***HOXB3* methylation level in groups of AML patients with and without *FLT3* mutations. **(g)***HOXB3* methylation level in groups of AML patients with and without *DNMT3A* mutations. **(h)***HOXB3* methylation level in groups of AML patients with and without *RUNX1* mutations. The differences between groups were determined by Mann-Whitney U test/Kruskal-Wallis test.

Based on the dramatical clinical relations, we further investigated the *HOXB3* methylation level in AML patients among different FAB subtypes, karyotypic classifications, and molecular/cytogenetic risks. Expectedly, significant differences were confirmed in the FAB subtypes (*P* = 0.004), karyotypic classifications (*P* < 0.001), and molecular/cytogenetic risks (both *P* < 0.001) (**Figures 2a–d**). Next, we explored *HOXB3* methylation levels in AML patients with and without *NPM1*/*FLT3*/*DNMT3A*/*RUNX1* gene mutations. Similar results were observed in *NPM1* (*P* < 0.001), *FLT3* (*P* < 0.001), *DNMT3A* (*P* < 0.001) mutation and *RUNX1* wild-type (*P* = 0.009) (**Figures 2e–h**).

### The independent prognostic value of HOXB3 hypomethylation in AML

Since a close correlation was found between *HOXB3* hypo-methylation and common prognostic factor karyotypic classifications, we further performed multivariate Cox regression to confirm the independent prognostic impact of *HOXB3* hypo-methylation in AML after adjusting for prognosis-related factors. Cox regression multivariate analysis indicated that *HOXB3* expression was an independent risk factor affecting OS and DFS in both total AML (*P* = 0.023 and 0.009, respectively) and non-M3 AML (*P* = 0.024 and 0.039, respectively) patients (**Table 3**).

**Table 3:** Univariate and multivariate Cox regression analysis of potential risk factors for overall survival and leukemia free survival in AML patients.

Variables	Total AML				Non-M3 AML			
	Univariate analysis		Multivariate analysis		Univariate analysis		Multivariate analysis	
	HR (95% CI)	*P*	HR (95% CI)	*P*	HR (95% CI)	*P*	HR (95% CI)	*P*
Overall survival								
Age	1.039 (1.026-1.053)	<0.001	1.026 (1.012-1.040)	<0.001	1.032 (1.018-1.046)	<0.001	1.020 (1.005-1.035)	0.007
WBC	1.003 (0.999-1.006)	0.119	1.002 (0.999-1.006)	0.150	1.001 (0.997-1.005)	0.558		
Molecular risks	1.870 (1.474-2.371)	<0.001	2.246 (1.704-2.962)	<0.001	1.701 (1.303-2.222)	<0.001	2.173 (1.610-2.934)	<0.001
Treatment regimen	0.540 (0.380-0.767)	0.001	0.479 (0.319-0.720)	<0.001	0.446 (0.311-0.639)	<0.001	0.432 (0.286-0.654)	<0.001
*FLT3* mutation	1.271 (0.870-1.858)	0.215			1.352 (0.915-2.000)	0.130	1.423 (0.932-2.174)	0.102
*NPM1* mutation	1.220 (0.836-1.780)	0.302			1.063 (0.725-1.557)	0.756		
*DNMT3A* mutation	1.615 (1.100-2.373)	0.015	1.153 (0.751-1.771)	0.514	1.431 (0.971-2.109)	0.070	1.147 (0.749-1.756)	0.528
*HOXB3* methylation	0.613 (0.432-0.869)	0.006	0.664 (0.467-0.945)	0.023	0.620 (0.433-0.890)	0.009	0.656 (0.455-0.946)	0.024
Disease-free survival								
Age	1.034 (1.021-1.047)	<0.001	1.024 (1.010-1.038)	0.001	1.027 (1.013-1.040)	<0.001	1.021 (1.007-1.036)	0.004
WBC	1.003 (1.000-1.006)	0.094	1.002 (0.999-1.006)	0.139	1.001 (0.998-1.005)	0.422		
Molecular risks	1.796 (1.424-2.265)	<0.001	2.040 (1.563-2.661)	<0.001	1.625 (1.253-2.109)	<0.001	2.005 (1.503-2.675)	<0.001
Treatment regimen	0.607 (0.427-0.862)	0.005	0.565 (0.380-0.839)	0.005	0.522 (0.365-0.748)	<0.001	0.532 (0.357-0.792)	0.002
*FLT3* mutation	1.252 (0.857-1.829)	0.245			1.343 (0.909-1.983)	0.139	1.442 (0.944-2.201)	0.090
*NPM1* mutation	1.263 (0.865-1.843)	0.227			1.107 (0.755-1.622)	0.604		
*DNMT3A* mutation	1.524 (1.040-2.235)	0.031	1.059 (0.691-1.624)	0.792	1.360 (0.924-2.001)	0.119	1.059 (0.692-1.620)	0.793
*HOXB3* methylation	0.608 (0.429-0.862)	0.005	0.627 (1.563-2.661)	0.009	0.621 (0.433-0.890)	0.009	0.671 (0.460-0.980)	0.039

HR, hazard ratio; CI, confidence interval; WBC, white blood cells. Variables including age (continuous variables), WBC (continuous variables), treatment regimen (with transplantation vs. without transplantation), molecular risks (good, intermediate, poor and unknown; classified by the 2017 European LeukemiaNet classification), *FLT3*/*NPM1*/*DNMT3A* mutation (wild type vs. mutant) and *HOXB3* methylation (hyper- vs. hypo-). Multivariate analysis includes variables with *P* < 0.200 in univariate analysis.

### Molecular alterations correlated with HOXB3 dysregulation in AML

**Figure 3:**
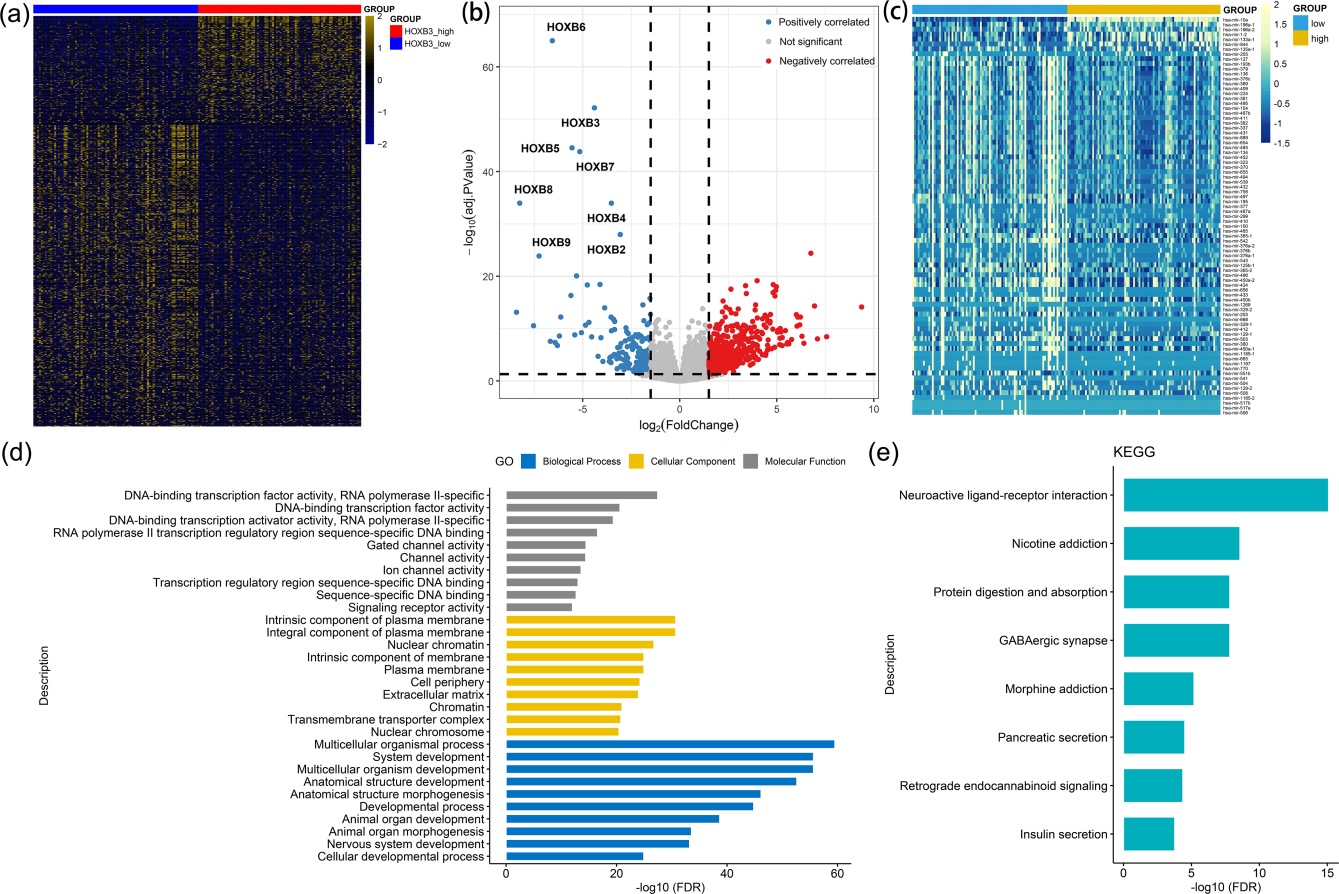
Molecular signatures associated with *HOXB3* expression in AML. **(a)** Expression heatmap of differentially expressed genes including mRNAs/lncRNAs (DEGs) between low and high *HOXB3* expression groups in AML (|log2 FC|>1.5, FDR<0.05 and *P* < 0.05). **(b)** Volcano plot of DEGs between low and high *HOXB3* expression groups in AML. **(c)** Expression heatmap of differentially expressed miRNAs between low and high *HOXB3* expression groups in AML (|log2 FC|>1.5, FDR<0.05 and *P* < 0.05). **(d)** Gene Ontology (GO) and Kyoto Encyclopedia of Genes and Genomes (KEGG) analysis of above DEGs.

To investigate the biological network correlated with *HOXB3* dysregulation in AML, we studied the transcriptomes of AML samples with low and high *HOXB3* expression from TCGA database. A total of 784 differentially expressed genes (DEGs, including mRNAs and long noncoding RNAs) (208 positively correlated and 576 negatively correlated) and 81 differentially expressed miRNAs (DEmiRs) (8 positively correlated and 73 negatively correlated) were identified between the two groups (**Figures 3a–c**; **Supplementary Table S2**). The positively correlated DEGs (Top 10) were *HOXB* family members, which were reported to have pro-leukemic effects and were associated with clinical outcomes of AML (8–10, 15). Moreover, positively correlated DEmiRs (top 5) such as *miR-10*, *miR-196a* and *miR-1* were reported to have pro-leukemic effects (16–18), whereas negatively correlated DEmiRs (top 5) such as *miR-193b* and *miR-379* were reported to have anti-leukemic effects in AML (19, 20). Moreover, Gene Ontology (GO) analysis demonstrated that these DEGs were involved in multiple biological processes such as development (**Figure 3d**).

### Validation of HOXB3 hypomethylation and its epigenetically regulatory role in AML

**Figure 4:**
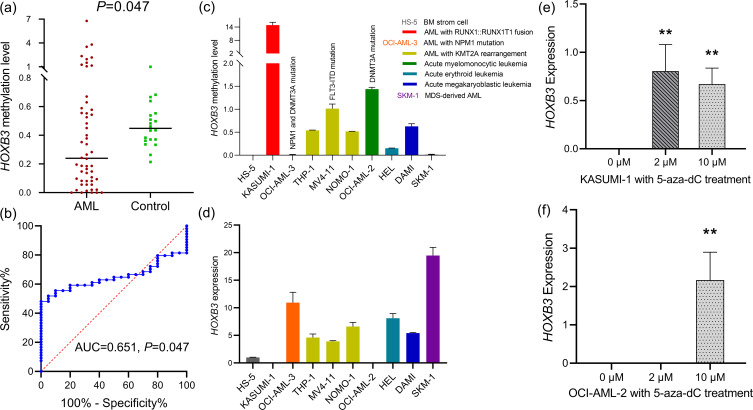
Validation of aberrant *HOXB3* hypomethylation and its epigenetically regulatory role in AML. **(a)***HOXB3* methylation in AML patients from our hospital. **(b)** Receiver operating characteristic curve analysis of *HOXB3* methylation in distinguishing AML patients from controls. **(c)***HOXB3* methylation in AML cell lines. **(d)***HOXB3* expression in AML cell lines. **(e)***HOXB3* expression in KASUMI-1 cell-line after 5-aza-dC treatment at different dose. **(f)***HOXB3* expression in OCI-AML-2 cell-line after 5-aza-dC treatment at different dose. The differences between groups were determined by Mann-Whitney U test and Student T test. *P < 0.05; **P < 0.01; ***P < 0.001; ns: no significant.

To validate epigenetic dysregulation of *HOXB3* during leukemogenesis, we first determined *HOXB3* methylation levels in *de novo* AML and controls from the validation cohort. *HOXB3* methylation levels in AML patients were markedly reduced compared with those in the controls (*P* = 0.047, **Figure 4a**). ROC curve analysis indicated that *HOXB3* methylation might act as a potential biomarker for distinguishing AML patients from controls, with an AUC of 0.651 (95% CI: 0.532-0.770, *P* = 0.047) (**Figure 4b**). Next, we detected the methylation and expression of *HOXB3* in nine AML cell lines and one BM stoma cell line. KASUMI-1 and OCI-AML-2 showed the highest *HOXB3* methylation levels with a relatively low *HOXB3* expression levels, whereas OCI-AML-3 and SKM-1 presented the lowest *HOXB3* methylation levels with a relatively high *HOXB3* expression levels (**Figure 4c-d**). *HOXB3* expression showed a nearly negative correlation with *HOXB3* methylation in nine AML cell lines and one BM stoma cell line (R=-0.612, *P* = 0.067), indicating that *HOXB3* methylation plays a vital role in regulating *HOXB3* expression. To confirm the epigenetic regulatory role, KASUMI-1 and OCI-AML-2 cell lines were treated with the hypomethylation agent 5-aza-dC. Expectedly, *HOXB3* expression levels were markedly increased after 5-aza-dC treatment (**Figures 4e-f**).

## Discussion

Accumulating evidence has demonstrated aberrant *HOX* gene expression in diverse blood cancers, especially AML, but the epigenetic regulatory mechanism in AML remains largely elusive (5). Previously, we systematically identified the prognostic value of *HOXA* family gene methylation in AML, and revealed *HOXA9* hypomethylation is an epigenetic biomarker for predicting clinical outcomes and guiding treatment choice in AML (7). Herein, we further determined *HOXB* family genes methylation in AML, and revealed *HOXB3* hypomethylation as an epigenetic biomarker independently affects clinical outcome in AML. Similarly, Lindblad et al. reported that *HOXB2* and *HOXB3* expressions are independent prognostic markers in AML (9). As we have also demonstrated the epigenetic regulatory role of *HOXB3* methylation in AML, we propose that the prognostic value and functional role of *HOXB3* hypomethylation in AML mainly depends on its expression. In accordance with our study, Qu et al. showed that decreased methylation of *HOXB3* and *HOXB4* is associated with increased expression of both *HOXB* genes specific to mid-risk AML (21). These results suggest *HOXB3* methylation and expression are potential biomarkers for AML in clinical use.

Distinct patterns of *HOX* family gene expression have been described in defined cytogenetic and molecular subsets of patients with AML. Drabkin et al. reported that favorable and poor prognostic chromosomal rearrangements in AML were associated with distinct levels of HOX expression (22). Moreover, *HOX* overexpression may be correlated with a distinct biologic subset of AML with intermediate cytogenetics (23). Consistently, we observed a marked associations of *HOXB3* hypomethylation with FAB subtypes (M5), different karyotypes (normal karyotype), and molecular/cytogenetic risk classifications (intermediate risk). Moreover, we also found that *HOXB3* hypomethylation correlated with *NPM1*, *FLT3*, and *DNMT3A* mutations. Similarly, previous studies have indicated that the high expression of several *HOX* genes is associated with *NPM1*, *FLT3* and *DNMT3A* mutations (23–26). Moreover, several studies also addressed the closely associations of aberrant *HOXB* expression/methylation with *NPM1*, *FLT3*, and *DNMT3A* mutations in AML (10, 21, 27). These results indicated that *HOXB3* is correlated with certain genetic contexts in AML and may be a common downstream gene with potential targets in these specific AML subtypes.

*HOX* family members have been identified as transcription factors that activate their own expression or cross-regulate their family members or their cofactors (4). In our study, we also observed that *HOXB3* is closely related to the expression of other *HOX* family genes enriched in the same AML-related function. Moreover, several leukemia-related miRNAs, such as *miR-10*, *miR-196a*, *miR-1*, *miR-193b* and *miR-379*, have also been found to be associated with *HOXB3* dysregulation (16–20). Consistently, Chen et al. found that *HOXB5* is closely associated with the expression of other *HOX* family genes (10), which supports our results.

Although our study yielded significant results, it has some limitations in the current research. First, the clinical implications of *HOXB3* hypomethylation in AML were drawn from public databases and validated using only a small cohort of AML patients from our hospital. These results need to be verified in a larger cohort of AML patients. Second, few functional and mechanical studies have determined the underlying role of *HOXB3* hypomethylation in AML. Future studies should incorporate these experiments in order to further corroborate our research findings.

## Conclusion

Taken together, *HOXB3* hypomethylation, which is associated with *HOXB3* overexpression, is a frequent event in AML. AML with *HOXB3* hypomethylation usually has unique genetic patterns such as a normal karyotype, cytogenetic/molecular-intermediate risk, and mutations in *FLT3*-ITD, *NPM1* and *DNMT3A*. Despite these associations, *HOXB3* hypomethylation may serve as an independent prognostic biomarker for AML.

## Data Availability

The original contributions presented in the study are included in the article/**Supplementary Material**. Further inquiries can be directed to the corresponding author.

## References

[1] DiNardo CD Erba HP Freeman SD Wei AH . Acute myeloid leukaemia. Lancet. (2023) 401:2073–86. doi: 10.1016/S0140-6736(23)00108-3, PMID: 37068505

[2] Snaith O Poveda-Rogers C Laczko D Yang G Morrissette JJD . Cytogenetics and genomics of acute myeloid leukemia. Best Pract Res Clin Haematol. (2024) 37:101533. doi: 10.1016/j.beha.2023.101533, PMID: 38490763

[3] Estey EH . Epigenetics in clinical practice: the examples of azacitidine and decitabine in myelodysplasia and acute myeloid leukemia. Leukemia. (2013) 27:1803–12. doi: 10.1038/leu.2013.173, PMID: 23757301

[4] Alharbi RA Pettengell R Pandha HS Morgan R . The role of HOX genes in normal hematopoiesis and acute leukemia. Leukemia. (2013) 27:1000–8. doi: 10.1038/leu.2012.356, PMID: 23212154

[5] Collins CT Hess JL . Role of HOXA9 in leukemia: dysregulation, cofactors and essential targets. Oncogene. (2016) 35:1090–8. doi: 10.1038/onc.2015.174, PMID: 26028034 PMC4666810

[6] Roux B Picou F Debeissat C Koubi M Gallay N Hirsch P . Aberrant DNA methylation impacts HOX genes expression in bone marrow mesenchymal stromal cells of myelodysplastic syndromes and de novo acute myeloid leukemia. Cancer Gene Ther. (2022) 29:1263–75. doi: 10.1038/s41417-022-00441-w, PMID: 35194200

[7] Xie F Zhang TJ Zhang XL Xu ZJ Qiao L Wang Y . Identification of HOXA9 methylation as an epigenetic biomarker predicting prognosis and guiding treatment choice in acute myeloid leukemia. BMC Cancer. (2025) 25:215. doi: 10.1186/s12885-025-13633-y, PMID: 39920624 PMC11806540

[8] Umeda S Yamamoto K Murayama T Hidaka M Kurata M Ohshima T . Prognostic significance of HOXB4 in *de novo* acute myeloid leukemia. Hematology. (2012) 17:125–31. doi: 10.1179/102453312X13376952196250, PMID: 22664110

[9] Lindblad O Chougule RA Moharram SA Kabir NN Sun J Kazi JU . The role of HOXB2 and HOXB3 in acute myeloid leukemia. Biochem Biophys Res Commun. (2015) 467:742–7. doi: 10.1016/j.bbrc.2015.10.071, PMID: 26482852

[10] Chen M Qu Y Yue P Yan X . The prognostic value and function of HOXB5 in acute myeloid leukemia. Front Genet. (2021) 12:678368. doi: 10.3389/fgene.2021.678368, PMID: 34421991 PMC8376581

[11] Cancer Genome Atlas Research Network Ley TJ Miller C Ding L Raphael BJ Mungall AJ . Genomic and epigenomic landscapes of adult *de novo* acute myeloid leukemia. N Engl J Med. (2013) 368:2059–74. doi: 10.1056/NEJMoa1301689, PMID: 23634996 PMC3767041

[12] Cerami E Gao J Dogrusoz U Gross BE Sumer SO Aksoy BA . The cBio cancer genomics portal: an open platform for exploring multidimensional cancer genomics data. Cancer Discov. (2012) 2:401–4. doi: 10.1158/2159-8290.CD-12-0095, PMID: 22588877 PMC3956037

[13] Zhou JD Zhao YJ Leng JY Gu Y Xu ZJ Ma JC . DNA methylation-mediated differential expression of DLX4 isoforms has opposing roles in leukemogenesis. Cell Mol Biol Lett. (2022) 27:59. doi: 10.1186/s11658-022-00358-0, PMID: 35883028 PMC9327205

[14] Zhang TJ Xu ZJ Wen XM Gu Y Ma JC Yuan Q . SLIT2 promoter hypermethylation-mediated SLIT2-IT1/miR-218 repression drives leukemogenesis and predicts adverse prognosis in myelodysplastic neoplasm. Leukemia. (2022) 36:2488–98. doi: 10.1038/s41375-022-01659-1, PMID: 35906386

[15] Kusakabe M Sun AC Tyshchenko K Wong R Nanda A Shanna C . Synthetic modeling reveals HOXB genes are critical for the initiation and maintenance of human leukemia. Nat Commun. (2019) 10:2913. doi: 10.1038/s41467-019-10510-8, PMID: 31266935 PMC6606637

[16] Havelange V Ranganathan P Geyer S Nicolet D Huang X Yu X . Implications of the miR-10 family in chemotherapy response of NPM1-mutated AML. Blood. (2014) 123:2412–5. doi: 10.1182/blood-2013-10-532374, PMID: 24596420 PMC3983615

[17] Fan B Wang L Hu T Zheng L Wang J . Exosomal miR-196a-5p secreted by bone marrow mesenchymal stem cells inhibits ferroptosis and promotes drug resistance of acute myeloid leukemia. Antioxid Redox Signal. (2025) 42:933–53. doi: 10.1089/ars.2024.0882, PMID: 40388337

[18] Ghazaryan A Wallace JA Tang WW Barba C Lee SH Bauer KM . miRNA-1 promotes acute myeloid leukemia cell pathogenesis through metabolic regulation. Front Genet. (2023) 14:1192799. doi: 10.3389/fgene.2023.1192799, PMID: 37229187 PMC10203238

[19] Bhayadia R Krowiorz K Haetscher N Jammal R Emmrich S Obulkasim A . Endogenous tumor suppressor microRNA-193b: therapeutic and prognostic value in acute myeloid leukemia. J Clin Oncol. (2018) 36:1007–16. doi: 10.1200/JCO.2017.75.2204, PMID: 29432078

[20] Wu H Zhao L Guo H Xie Y Hu J Tan X . miR-379-5p inhibited the proliferation of acute myeloid leukemia cells through negative regulation of YBX1. Turk J Haematol. (2025) 42:92–9. doi: 10.4274/tjh.galenos.2025.2024.0424, PMID: 39992162 PMC12099468

[21] Qu X Davison J Du L Storer B Stirewalt DL Heimfeld S . Identification of differentially methylated markers among cytogenetic risk groups of acute myeloid leukemia. Epigenetics. (2015) 10:526–35. doi: 10.1080/15592294.2015.1048060, PMID: 25996682 PMC4623036

[22] Drabkin HA Parsy C Ferguson K Guilhot F Lacotte L Roy L . Quantitative HOX expression in chromosomally defined subsets of acute myelogenous leukemia. Leukemia. (2002) 16:186–95. doi: 10.1038/sj.leu.2402354, PMID: 11840284

[23] Roche J Zeng C Barón A Gadgil S Gemmill RM Tigaud I . Hox expression in AML identifies a distinct subset of patients with intermediate cytogenetics. Leukemia. (2004) 18:1059–63. doi: 10.1038/sj.leu.2403366, PMID: 15085154

[24] Andreeff M Ruvolo V Gadgil S Zeng C Coombes K Chen W . HOX expression patterns identify a common signature for favorable AML. Leukemia. (2008) 22:2041–7. doi: 10.1038/leu.2008.198, PMID: 18668134 PMC2676170

[25] Brunetti L Gundry MC Sorcini D Guzman AG Huang YH Ramabadran R . Mutant NPM1 maintains the leukemic state through HOX expression. Cancer Cell. (2018) 34:499–512.e9. doi: 10.1016/j.ccell.2018.08.005, PMID: 30205049 PMC6159911

[26] Tan YT Sun Y Zhu SH Ye L Zhao CJ Zhao WL . Deregulation of HOX genes by DNMT3A and MLL mutations converges on BMI1. Leukemia. (2016) 30:1609–12. doi: 10.1038/leu.2016.15, PMID: 26854025

[27] Liu HC Shih LY May Chen MJ Wang CC Yeh TC Lin TH . Expression of HOXB genes is significantly different in acute myeloid leukemia with a partial tandem duplication of MLL vs. a MLL translocation: a cross-laboratory study. Cancer Genet. (2011) 204:252–9. doi: 10.1016/j.cancergen.2011.02.003, PMID: 21665178

