# Supplementary material for: Identification and validation of HOXB3 hypomethylation as a novel prognostically epigenetic biomarker in acute myeloid leukemia
Source: Front Immunol. 2026 Jan 12;16:1709417. doi: 10.3389/fimmu.2025.1709417 (PMC12832762; doi:10.3389/fimmu.2025.1709417)
Supplement: Supplementary file 1 [file Table1.docx]

**Table S1. Clinic-pathologic characteristics of AML patients from our research center**

|  | Patient's parameters (n=54) |
| --- | --- |
| Sex, male/female | 30/24 |
| Median age, years (range) | 51.5 (18-81) |
| Median WBC, ×10^9^/L (range) | 18.95 (0.8-528.0) |
| Median hemoglobin, g/L (range) | 77 (53-138) |
| Median platelets, ×10^9^/L (range) | 50 (3-264) |
| BM blasts, % (range) | 46.25 (5.5-97.5) |

AML: acute myeloid leukemia; WBC: white blood cells; BM: bone marrow. *: AML patients less than 20% BM blasts often with typical cytogenetics such as t(8;21), t(16;16) and t(15;17) etc.
